# Comment on “Humidification with High-Flow Nasal Cannula and Airway Epithelial Cells: Caution, Still Learning from an Extremely Complex Environment”

**DOI:** 10.1155/2012/368713

**Published:** 2012-12-24

**Authors:** Antonio M. Esquinas, Naomi Kondo Nakagawa, Luiz Fernando Ferraz Da Silva

**Affiliations:** ^1^Intensive Care Unit, Hospital Morales Meseguer, Avenida Marques Velez s/n, 30008 Murcia, Spain; ^2^Department of Physiotherapy, Communication Science and Disorders and Occupational Therapy, Faculdade de Medicina da Universidade de São Paulo, Avenida Dr. Arnaldo, 455 São Paulo, SP, Brazil; ^3^Departamento de Patologia, Faculdade de Medicina da Universidade de São Paulo, São Paulo, SP, Brazil

We read with interest the paper by Chidekel et al. [1] on the effects of gas humidification with high-flow nasal cannula on cultured human airway epithelial cells. The authors used an “*in vitro*” method of bronchial epithelial cells culture and found increased total protein secretion and secretion of interleukins 6 and 8 after 8 hours of dry airflow compared with 69% and >90% of relative humidification groups as well as histological injury. 

The results raise the interest of this area focusing on the effects of humidification during spontaneous breathing and mechanical ventilation on epithelial cell structure, function and inflammatory response as pointed out by the authors [2, 3]. We are aware that all several variables that may interfere in this physiological process cannot be completely reproducible and controlled by *in vitro* models; however, some interesting aspects need attention.

1. The observational chamber ideally reflects nasal nares with a double entry that allows separation of the flow and resistance. We know that physiologically the airflow that would across the mucosa may be lower, histological changes observed could be different, and serious deleterious to the nasal cells might be more delayed in their presentation. For instance, pediatric patients are more vulnerable, and these mechanisms may be more or less obvious or surprising [4].
2. During expiration, heat and humidity (water vapour content) are partially retained in the nasopharynx region (“rewarming phenomenon”). From the clinical point of view, this is a critical aspect in physiological air conditioning that may be considered in an experimental “*in vitro*” model [5].
3. There are several articles describing the effects of positive pressure on respiratory epithelium, including some from the same group. These studies show that the high flow, the oxygenation, and the positive pressure induce considerable nasal epithelium changes [6]. However, it is still unknown if the constant pressure flow at cell culture chamber has some influence and should be taken into account when evaluating such epithelial changes.

We know that the nasal mucosa has a high capacity of vasodilatation which allows adjustment of the heating and humidification. Some of the observed effects in the related study may be partially explained by the absence of this factor. This is an example of a variable that cannot be addressed using such “*in vitro*” models.

The physiological airway flow is not continuous, but it has turbulences allowing moisture uptake, what is not the case in the presented study, and also may explain some of the observed changes [6]. One applicable possibility to simulate these variances in “*in vitro*” models is to vary the amount of flow during the experiments.

The authors attest that humidification therapy reduces airway epithelial inflammation compared to cells exposed to a dry condition over an 8-hour exposure time [1]. We believe that is inverse. The physiological pattern is to have adequate conditions of humidity and heating, thus the exposure to dry air induces deleterious and inflammatory effects on epithelium.

In conclusion, this interesting experimental model is important and plausible to investigate the effects of humidification and airflow on epithelial cells structure, function and inflammatory response, but includes some limitations lacking some physiological factors that may, indeed, affect the respiratory epithelial cells during spontaneous breathing and mechanical ventilation when considering the translation from experimental models to bedside.
